# The causal effect of matrix metalloproteinase-3 on ankylosing spondylitis: Evidence from Mendelian randomization

**DOI:** 10.1097/MD.0000000000041373

**Published:** 2025-01-31

**Authors:** Wenkai Liu, Licheng Guo, Yongfa Zhang, Wubing He

**Affiliations:** a Department of Emergency, Shengli Clinical Medical College of Fujian Medical University, Fujian Provincial Hospital, Fuzhou, Fujian, China; b Department of Emergency, Fuzhou University Affiliated Provincial Hospital, Fuzhou, Fujian, China.

**Keywords:** ankylosing spondylitis, causality, matrix metalloproteinase-3, Mendelian randomization

## Abstract

Previous investigations through observation have found that matrix metalloproteinase-3 (MMP-3) has benefits for ankylosing spondylitis (AS) but it is uncertain whether there is a true positive causal connection. Our goal was to demonstrate the relationship between AS and MMP-3. We executed Mendelian randomization (MR) research utilizing genome-wide association studies genetic data (n = 21,758) for MMP-3 publicly available from IEU Open and genome-wide association studies data for AS (n = 297,932) from FinnGen Biobank. The specific MR protocols were weighted median, weighted mode, MR-Egger, and inverse-variance weighted (IVW). Subsequently, the Cochran *Q* evaluate, MR pleiotropy residual sum and outlier, and MR-Egger intercept were used to evaluate the heterogeneity and multiplicative effects of instrumental variables. The IVW method demonstrated that MMP-3 had a causal effect on AS (odds ratio, 0.9047 [95% confidence interval, 0.8080–1.0129]; *P* = .0823). Certainly, other MR techniques were in accordance with the tendency of the IVW method (*P* < .05), and sensitivity testing verified the reliability of this MR result. This MR study substantiates the causal role of MMP-3 in the development of AS, offering valuable insights into the disease mechanism and potential therapeutic targets.

## 1. Introduction

Ankylosing spondylitis (AS) is a chronic inflammatory immunological disorder characterized by its severe manifestation and involvement of the axial skeleton, leading to progressive structural damage and functional impairments.^[1]^ The etiopathogenesis of AS is intricate and multifactorial, involving an intricate interplay between genetic predispositions and environmental triggers.^[2]^ Among the myriad of potential contributors, matrix metalloproteinase-3 (MMP-3) has emerged as a molecule of profound interest due to its hypothesized involvement in the complex pathophysiological mechanisms underlying AS.^[3,4]^

MMP-3, also known as stromelysin-1, is a matrix-degrading enzyme endowed with the capacity to cleave and remodel various extracellular matrix components, including proteoglycans, fibronectin, and collagen.^[5]^ Remarkably, consistently elevated concentrations of MMP-3 have been detected in the serum and synovial fluid of patients with AS, lending credence to its potential role in the inflammatory cascades and structural alterations that characterize the disease.^[3,6,7]^ A plethora of observational studies have documented associations between MMP-3 levels or genetic variants and various facets of AS, such as susceptibility, disease activity, and radiographic progression.^[3,7]^ Nonetheless, these investigations are intrinsically restricted by the possibility of confounding elements and the inability to definitively establish the directionality of the observed associations, thereby hindering the unequivocal determination of a causal relationship between MMP-3 and AS.

Mendelian randomization (MR) is a strong epidemiological method that uses genetic variants to investigate exposure-outcome correlations.^[8,9]^ By capitalizing on the random assortment of genetic variants during gamete formation, MR analyses yield stronger proof of causality because they are very resistant to reverse causation and confounding variables.^[8]^

We used a 2-sample MR technique in this important study to thoroughly assess the causal relationship between MMP-3 and AS risk. We wanted to use summary-level data from large-scale, well-powered genome-wide association studies (GWAS) and several MR approaches to investigate the potential causative involvement of MMP-3 in the progress of AS. Our findings hold the promise of contributing to a more profound understanding of the disease mechanisms and potentially informing the growth of novel therapeutic strategies.

## 2. Materials and methods

### 2.1. Data sources

The exposure data for MMP-3 levels were obtained from a large-scale GWAS conducted by Folkersen et al^[10]^ and made publicly available through the IEU Open GWAS database (dataset ID: ebi-a-GCST90012027). This GWAS included 21,758 individuals of European ancestry and identified 13,057,986 single-nucleotide polymorphisms (SNPs) associated with MMP-3 levels. The outcome data for AS were derived from the FinnGen Biobank GWAS study (dataset ID: finngen_R10_M13_ANKYLOSPON), which included 297,932 individuals of European ancestry and identified 16,380,022 SNPs associated with AS risk (Table 1 displays the data source information).

**Table 1:** Phenotype source and exposure and outcomes.

Phenotypes		ID	First author (yr)	Sample size	SNPs	Population
Exposure	Matrix metalloproteinase-3	ebi-a-GCST90012027	Lasse Folkersen (2020)	21,758	13,057,986	European
Outcomes	Ankylosing spondylitis	finngen_R10_M13_ANKYLOSPON	NA (2021)	297,932	16,380,022	European

SNP = single-nucleotide polymorphism.

### 2.2. MR analysis

This MR method leverages genetic variations used as instrumental variables (IVs) to overcome potential confounding factors and reverse causation by offering more comprehensive proof of causation. We identified independent genome-wide significant SNPs (*P* < 5 × 10^−8^) associated with MMP-3 levels as IVs After extracting the significant SNPs corresponding to MMP-3, a linkage disequilibrium analysis was performed, and the SNPs with the smallest *P* value were considered as independent genetic variants (linkage disequilibrium *r*^2^ < 0.001 and clumping window size = 10,000 kb). To assess the strength of each IV, we calculated the *F*-statistic for each SNP. *F* > 10 suggests the absence of weak instrumental bias. The detailed information on the IVs is listed in Table S1, Supplemental Digital Content, http://links.lww.com/MD/O311. These IVs were then used to estimate the causal impact of MMP-3 on AS risk using the following MR methods.^[8,9]^ Inverse-variance weighted (IVW): this approach integrates the ratio estimates obtained from individual SNPs into a single overall estimate, assuming no horizontal pleiotropy, which is currently the most statistically efficient MR method. MR-Egger regression: this method accounts for potential horizontal pleiotropy by enabling the regression model’s intercept, providing a consistent estimate of the causal effect even in the presence of pleiotropy. Weighted median estimator: this method yields a constant estimate of the causal influence even when up to 50% of the weight comes from erroneous IVs. Weighted mode: this approach is resistant to outliers and offers a reliable estimation of the causal effect, even in situations where the majority of the independent IVs are not true.

### 2.3. MR sensitivity testing

To assess the validity of the MR assumptions and the robustness of the causal estimates. Cochran *Q* statistic was used to evaluate the presence of heterogeneity among the ratio estimates from individual SNPs.^[11]^ In order to test for horizontal pleiotropy, the MR pleiotropy residual sum and outlier (MR-PRESSO) method was also utilized throughout the process.^[12,13]^ The intercept term from the MR-Egger regression was used to detect potential directional pleiotropy.^[14]^

### 2.4. Statistical analysis

All statistical analyses were performed using specialized MR software packages, such as *MR-Base, TwoSampleMR*, and *Mendelian Randomization*. All statistical analyses were conducted using R version 4.2.1 (Auckland, New Zealand). The study presented the causative estimates as odds ratios along with 95% confidence intervals (CIs). When the *P* value was <.05, it was deemed to be statistically valid, indicating evidence of a causal effect.

## 3. Results

### 3.1. MR results of MMP-3 and AS

The MR analysis results for the causal effect of MMP-3 on AS risk are presented in Table 2. Using the IVW method, which assumes no horizontal pleiotropy, we observed a suggestive causal effect of MMP-3 on AS (odds ratio, 0.9047 [95% CI, 0.8080–1.0129]; *P* = .0823). To account for potential horizontal pleiotropy, we employed additional MR methods, including MR-Egger regression, weighted median estimator, and MR-PRESSO. The results from these methods were consistent with the IVW findings, although with wider CIs due to the more stringent assumptions (Table 2).

**Table 2:** MR analysis results between matrix metalloproteinase-3 and ankylosing spondylitis (*P <*.05).

MR method	nSNPs	*β*	SE	OR (95% CI)	*P*
Inverse-variance weighted	7	0.0004	0.0577	0.9047 (0.8080–1.0129)	.0823
MR-Egger	7	0.0004	0.0980	0.8489 (0.7006–1.0286)	.1553
Weighted median	7	0.0002	0.0663	0.8987 (0.7892–1.0234)	.1072
Weighted mode	7	0.0002	0.0665	0.9020 (0.7917–1.0276)	.1721

All data are accurate to 4 decimal places.

CI = confidence interval, IVW = inverse-variance weighted, MR = Mendelian randomization, nSNP = single nucleotide polymorphism, OR = odds ratio, SE = standard error.

**Figure 1.**
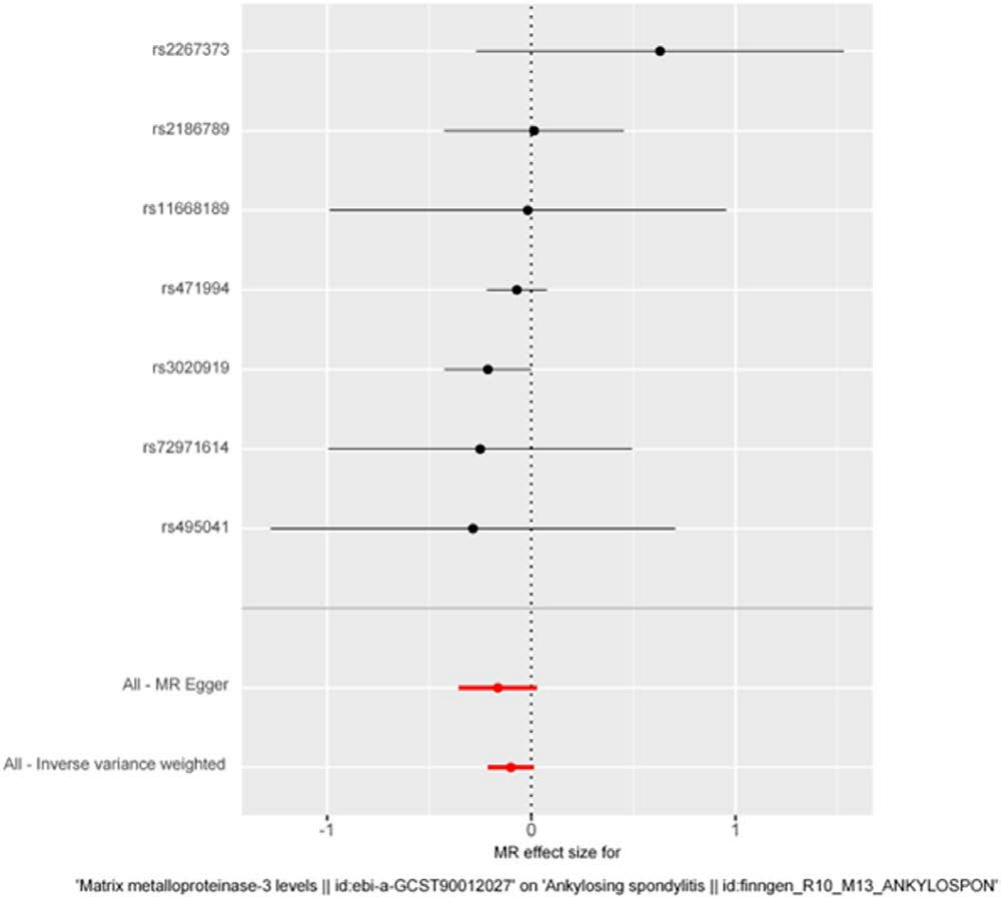
Forest plot of MR analysis for matrix metalloproteinase-3 and ankylosing spondylitis (*P* < .05). MR = Mendelian randomization.

**Figure 2.**
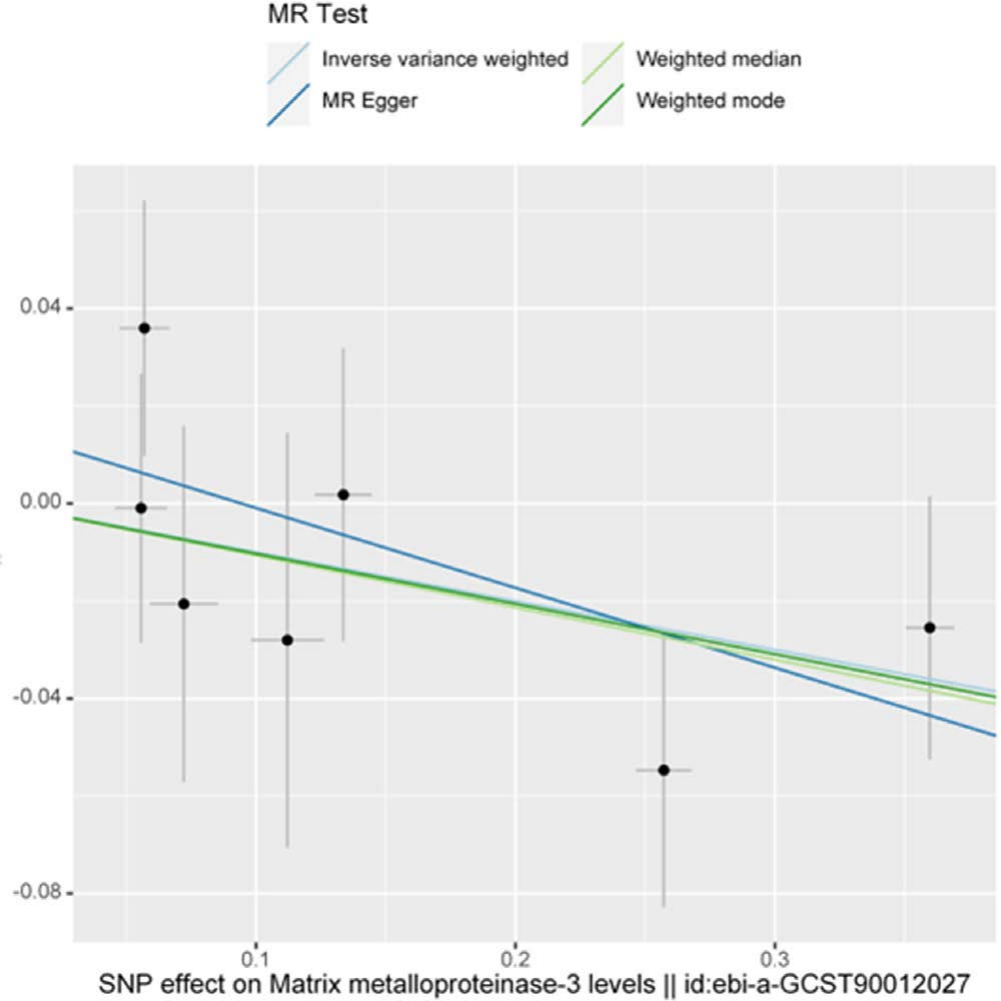
Scatter plots for MR analysis for matrix metalloproteinase-3 and ankylosing spondylitis. MR = Mendelian randomization, SNP = single-nucleotide polymorphism.

The forest plot in Figure 1 provides a visual representation of the causal estimates and their corresponding CIs obtained from the different MR methods. The scatter plots in Figure 2 illustrate the relationship between the genetic associations with MMP-3 and AS, providing an intuitive representation of the MR analysis.

### 3.2. Sensitivity analysis

To assess the validity of the MR assumptions and the robustness of the causal estimates, we performed several sensitivity analyses, as shown in Table 3. First, we evaluated the presence of heterogeneity among the ratio estimates from individual SNPs using Cochran *Q* statistic. The *Q* statistic of 4.3215 and a corresponding *P* value of .6332 indicated no significant heterogeneity, suggesting that the IVW assumption of consistent causal estimates across SNPs was not violated. Next, we employed the MR-Egger regression to detect potential directional pleiotropy. The intercept term from the MR-Egger analysis was not significantly different from zero (intercept = 0.0154; *P* = .4580), providing no evidence of directional pleiotropy biasing the causal estimate.

**Table 3:** Sensitivity analysis of the MR analysis results between matrix metalloproteinase-3 and ankylosing spondylitis (*P >*.05).

Exposure	Outcome	MR-Egger		Cochran *Q* test (IVW)		MR-PRESSO	
		Intercept value	*P*	*Q*	*P*	*T*-stat	*P*
Matrix metalloproteinase-3	Ankylosing spondylitis	0.0154	.458	4.3215	.6332	−2.0471	.0866

MR = Mendelian randomization, MR-PRESSO = MR pleiotropy residual sum and outlier.

**Figure 3.**
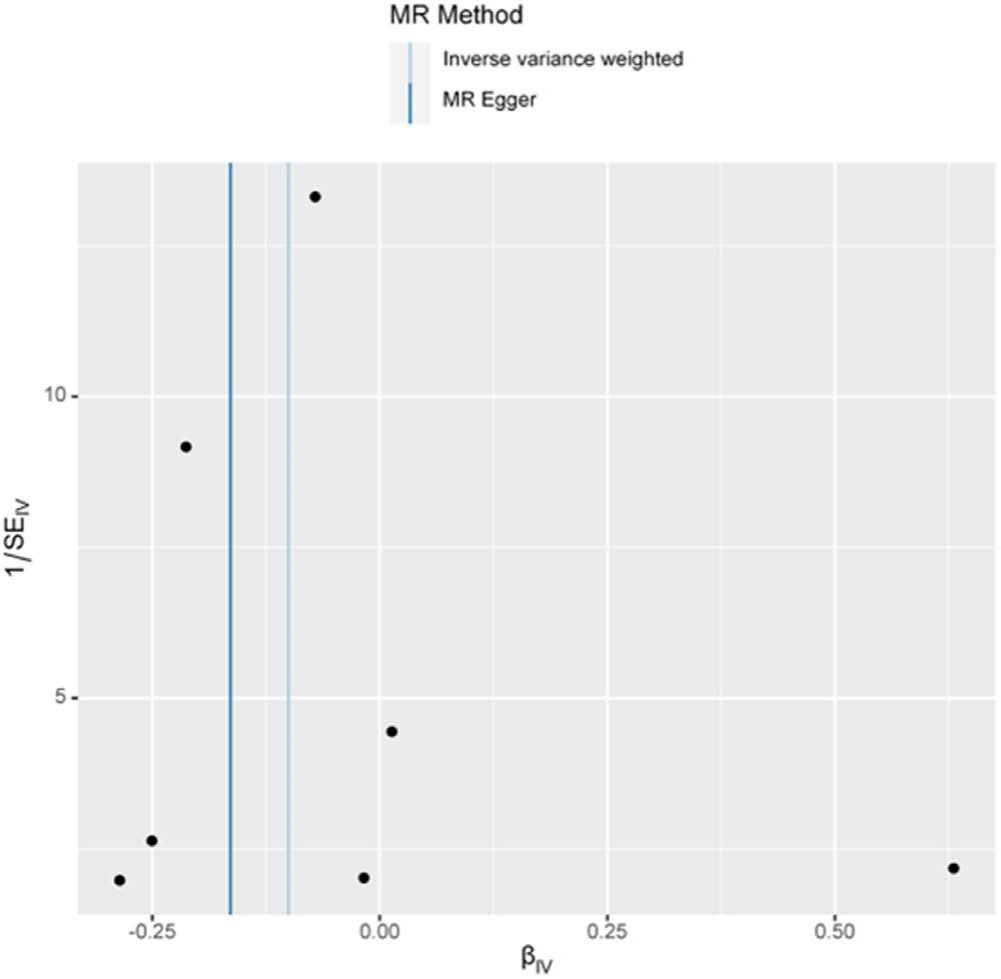
Funnel plot of MR analysis for matrix metalloproteinase-3 and ankylosing spondylitis. MR = Mendelian randomization.

In addition, we utilized the MR-PRESSO method to identify and correct for potential horizontal pleiotropy by removing outlying IVs from the analysis. The results of MR-PRESSO (*T*-stat = −2.0471; *P* = .0866) were consistent with the main MR findings, further supporting the robustness of the causal estimate. The funnel plot in Figure 3 provides a visual assessment of potential small-study bias or horizontal pleiotropy. The symmetrical distribution of the data points around the mean effect size suggests no significant bias or pleiotropy.

**Figure 4.**
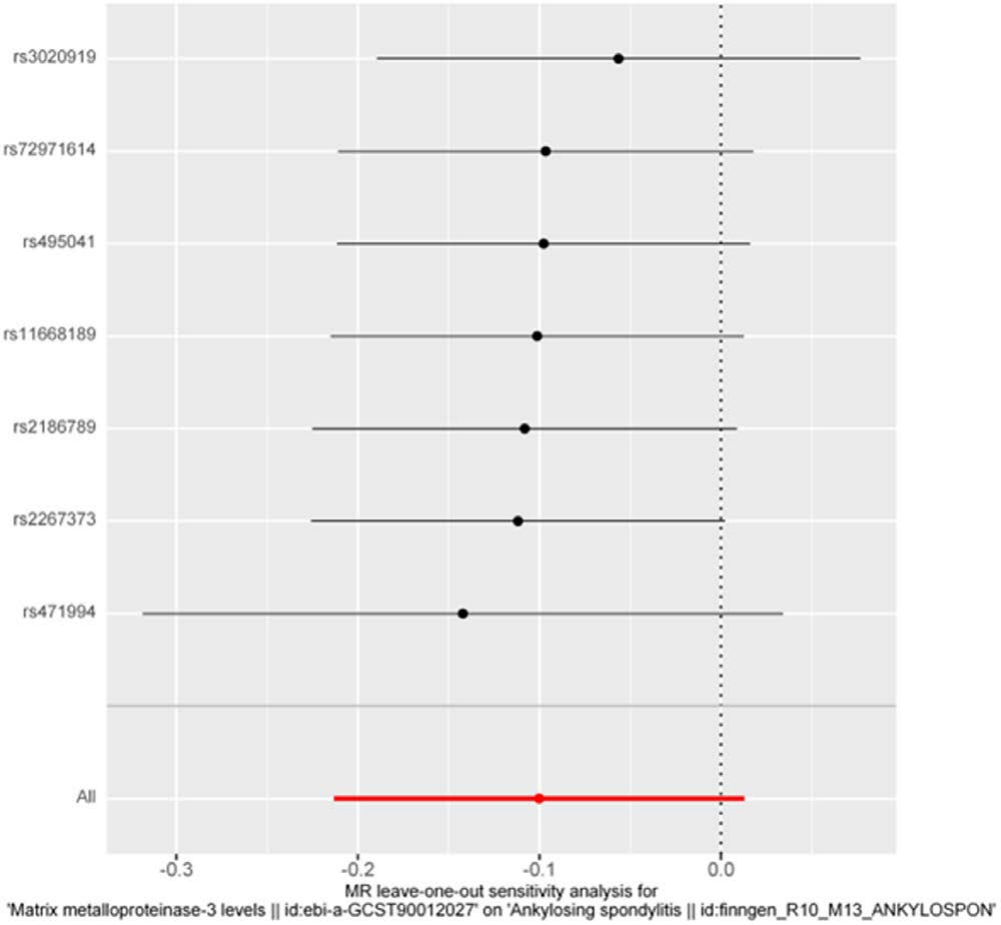
The leave-one-out analysis for matrix metalloproteinase-3 and ankylosing spondylitis.

Furthermore, we performed a leave-one-out analysis, as depicted in Figure 4, to evaluate the influence of individual SNPs on the overall causal estimate. The results showed no significant changes in the estimate when each SNP was iteratively removed, indicating that the causal effect was not driven by any single SNP.

Overall, the sensitivity analyses supported the validity of the MR assumptions and the robustness of the causal estimate, strengthening the evidence for a causal relationship between MMP-3 and AS risk.

## 4. Discussion

The present MR study provides robust evidence for a connection of causation between MMP-3 and AS risk. Utilizing genetic variants as instrumental elements allows for the following, we circumvented the limitations of traditional observational studies, such as confounding factors and reverse causation, to establish a more reliable causal inference.

Our results align with earlier observational research that has implicated MMP-3 in the pathogenesis of AS. Elevated levels of MMP-3 have been consistently observed in the serum and synovial fluid of patients with AS, suggesting its involvement in the inflammatory cascades and structural alteration characteristics of the disease.^[3,6,15]^ Furthermore, several studies have reported associations between MMP-3 genetic variants and various aspects of AS, including disease susceptibility, activity, and radiographic progression.^[7,16]^

The causal role of MMP-3 in AS can be attributed to its potent matrix-degrading properties. As a key member of the MMP family, MMP-3 is capable of cleaving and remodeling various extracellular matrix components, such as proteoglycans, fibronectin, and collagen.^[5,17]^ This enzymatic activity may contribute to the structural damage and joint erosions observed in patients with AS, particularly in the axial skeleton. Moreover, MMP-3 has been implicated in the modulation of inflammatory processes, which are central to the pathogenesis of AS. Elevated MMP-3 levels have been associated with increased production of proinflammatory cytokines, such as tumor necrosis factor-α.^[15,18]^ This proinflammatory milieu may perpetuate the chronic inflammation and tissue damage characteristic of AS.

Interestingly, our findings align with previous MR studies that have explored the causal relationships between other MMPs and AS. For instance, Xiao et al^[19]^ reported a causal effect of tissue inhibitor of metalloproteinase-3 on ischemic stroke and intracerebral hemorrhage, highlighting the potential role of MMPs in inflammatory and vascular processes.

The causal association between MMP-3 and AS risk revealed in this study has significant clinical implications. First, it provides mechanistic insights into the disease pathogenesis, potentially informing the development of novel therapeutic strategies targeting MMP-3 or its associated pathways. While current treatment options for AS, such as biologic agents like tumor necrosis factor-α inhibitors, have shown efficacy in managing disease activity and slowing radiographic progression,^[18]^ there remains a need for additional targeted therapies.

Furthermore, our findings suggest the potential utility of MMP-3 as a biomarker for disease monitoring and therapeutic response evaluation. Several studies have demonstrated the correlation between serum MMP-3 levels and disease activity, as well as its predictive value for treatment response.^[3,17,20]^ By incorporating MMP-3 measurements into clinical assessments, clinicians may gain valuable insights into disease progression and treatment efficacy, enabling more personalized and tailored management strategies.

It is noteworthy that our study is not without limitations. First, while the MR approach is robust against confounding factors and reverse causation, it cannot exclude the possibility of horizontal pleiotropy, where a genetic variant may influence the outcome through pathways independent of the exposure.^[4,21,22]^ However, our sensitivity analyses, including MR-Egger regression and MR-PRESSO, did not reveal significant horizontal pleiotropy, strengthening the validity of our findings. In addition, our study focused on genetic variants associated with MMP-3 levels, which may not fully capture the complex interplay between MMP-3 and other molecular players involved in the pathogenesis of AS. Future studies incorporating gene–environment interactions and epigenetic factors may provide a more comprehensive understanding of the disease mechanisms.

Despite these limitations, the present study contributes significantly to the understanding of the causal relationship between MMP-3 and AS risk. By employing a rigorous MR approach and leveraging large-scale GWAS data, we have provided robust evidence supporting the causal role of MMP-3 in the development of AS.

## 5. Conclusion

In conclusion, this MR study substantiates the causal effect of MMP-3 on AS risk. Our findings not only deepen the mechanistic insights into the disease pathogenesis but also highlight the potential of MMP-3 as a therapeutic target and biomarker for disease monitoring and treatment response evaluation. These findings pave the way for further exploration of MMP-3-targeted interventions and personalized management strategies for individuals with AS. Future research efforts should focus on validating these findings in diverse populations, elucidating the intricate molecular pathways involving MMP-3, and translating these insights into clinical applications for improved patient outcomes.

## Acknowledgments

The authors would like to express their gratitude to the volunteers and investigators involved in the FinnGen bioBank and the IEU GWAS project for their contributions to examining human medical research through their data.

## Author contributions

**Conceptualization:** Wenkai Liu.

**Writing – original draft:** Wenkai Liu.

**Data curation:** Licheng Guo.

**Methodology:** Licheng Guo.

**Formal analysis:** Yongfa Zhang.

**Investigation:** Yongfa Zhang.

**Funding acquisition:** Wubing He.

**Writing – review & editing:** Wubing He.

## Supplementary Material


